# Association between number of functional teeth and physical function among community-dwelling older adults: Korean Frailty and Aging Cohort Study

**DOI:** 10.1186/s12877-024-05585-y

**Published:** 2024-12-20

**Authors:** Nahyun Lim, Daehyun Lee, Seung-Yun Shin, Chang Won Won, Miji Kim

**Affiliations:** 1 Department of Precision Medicine, Graduate School, Kyung Hee University, Seoul, 02447 Republic of Korea; 2 KHU-KIST Department of Converging Science and Technology, Graduate School, Kyung Hee University, Seoul, 02447 Republic of Korea; 3 Department of Periodontology, Periodontal-Implant Clinical Research Institute, College of Dentistry, Kyung Hee University, Seoul, 02447 Republic of Korea; 4 Elderly Frailty Research Center, Department of Family Medicine, College of Medicine, Kyung Hee University, Seoul, 02447 Republic of Korea; 5 Department of Family Medicine, Kyung Hee University Medical Center, Seoul, 02447 Republic of Korea; 6 Department of Health Sciences and Technology, College of Medicine, Kyung Hee University, Seoul, 02447 Republic of Korea

**Keywords:** Functional teeth, Physical function, Older adults

## Abstract

**Background:**

Functional teeth are important for maintaining appropriate masticatory function and nutritional intake, affecting physical function in older adults. This study aimed to evaluate the association between number of functional teeth and physical function in community-dwelling older Korean adults.

**Methods:**

This cross-sectional study was conducted among a total of 2,527 participants (mean age, 76.5 ± 3.9; 53.4% women) who were enrolled in the Korean Frailty and Aging Cohort Study (2016–2017). Participants were categorized based on the number of functional teeth into two groups: < 20 and ≥ 20 functional teeth. Functional teeth were defined as the remaining natural teeth with visible crowns, with or without restorations, prosthetic pontics, or dental implants on panoramic radiography. Physical function was assessed using handgrip strength, gait speed, five-times sit-to-stand test (5TSTS) duration, and the Short Physical Performance Battery (SPPB) score. Multivariate linear and logistic regression models were used to evaluate the association between number of functional teeth and physical function.

**Results:**

Of all the participants, 869 (34.3%) had < 20 functional teeth. After full adjustment for sociodemographic factors, lifestyle, health condition, and oral health, an increase in one functional tooth was associated with a corresponding increase in gait speed (men: B = 0.002, *p* = 0.032; women: B = 0.002, *p* = 0.013) and SPPB (men: B = 0.019, *p* < 0.001; women: B = 0.018, *p* < 0.001) in both men and women. The time taken for the 5TSTS was shorter for an increase in one more functional tooth (men, B=-0.033, *p* = 0.006; women, B=-0.036, *p* = 0.021) in both men and women. An increase in one functional tooth was associated with a corresponding increase in handgrip strength only in men (men, B = 0.049, *p* = 0.009; women, B=-0.003, *p* = 0.814). The associations between < 20 functional teeth and low handgrip strength [odds ratio (OR) = 1.46, 95% confidence interval (CI): 1.03–2.06], long 5TSTS duration (OR = 1.47, 95% CI: 1.07–2.02), and low SPPB scores (OR = 1.64, 95% CI: 1.07–2.53) were significant in fully adjusted model compared with ≥ 20 functional teeth only in men.

**Conclusions:**

Fewer functional teeth were associated with low physical function in older adults. Our results emphasize the importance of maintaining adequate functional teeth to preserve physical function in community-dwelling older adults.

## Background

As physical function declines with age, it adversely affects the ability to maintain independent daily living [1] and is associated with frailty, disability, hospitalization, and mortality [2–5]. Therefore, maintaining physical function is important for older adults to live independently and remain healthy. Several studies have demonstrated that factors such as nutritional status, sleep quality, cognitive function, and chronic diseases [6–9] affect physical function. An inadequate nutrient intake induces a decline in muscle mass and function in older adults [6]. A previous study reported that older adults with impaired masticatory function often avoid raw vegetables and meat, leading to deficiencies in essential vitamins, minerals and protein [10]. Consequently, oral health status, which affects nutritional intake, influences muscle function in older adults. As tooth loss is generally used as the last indicator of oral health, previous studies have reported that having at least 20 natural teeth allows for maintaining normal masticatory function [11, 12]. Furthermore, the number of natural teeth is reportedly associated with sarcopenia and frailty, which are mediated by nutrition [13, 14] and physical function [15–17].

Functional teeth refer to teeth used for chewing food, including remaining natural teeth and prosthetically restored teeth [18, 19]. Given the involvement of these teeth in maintaining appropriate oral function, the importance of functional teeth has been increasing recently [20]. Prosthetic rehabilitation can increase the number of functional teeth in older adults with tooth loss. Indeed, in Korea, the national health insurance coverage for dental prosthetics for older adults has expanded since 2012. In 2019, 41.6% of older adults with total health insurance received treatment with dentures or dental implants at least once [21].

Recent studies on Japanese older adults showed that the number of functional teeth is a better predictor of all-cause mortality [19] and loss of independence [20] than the number of natural teeth. However, to the best of our knowledge, few studies have examined the association between the number of functional teeth and physical function in older adults. Furthermore, previous studies have reported inconsistent findings regarding the association between number of teeth and physical function. For instance, a study in Korea reported that a fewer number of remaining teeth was associated with low handgrip strength, whereas another study in Japan reported a non-significant association between the fewer number of teeth and low handgrip strength [22, 23]. In addition, it is reported that fewer number of teeth was associated with low handgrip strength only in men [17]. Furthermore, Musacchio et al. reported an association between the fewer number of teeth and both slow gait speed and lower Short Physical Performance Battery (SPPB) scores, whereas Viviana et al. observed no significant association between the fewer number of teeth and slow gait speed [24, 25]. However, studies examining this relationship according to sex for outcomes such as gait speed, five-times sit-to-stand test (5TSTS), and SPPB are scarce. Therefore, this study aimed to evaluate the association between the number of functional teeth and various physical and functional outcomes, including handgrip strength, gait speed, 5TSTS, and SPPB, focusing on sex-related differences in community-dwelling older Korean adults.

## Methods

### Study population

This was a cross-sectional study of baseline data from the nationwide Korean Frailty and Aging Cohort Study (KFACS), a nationwide longitudinal research initiative targeting individuals aged 70–84 years [26]. The baseline assessment was conducted between 2016 and 2017. In the KFACS, participants were recruited from among older adults living in communities in urban, suburban, and rural areas nationwide in ten study centers across different regions covering different residential locations. Participants were recruited from diverse settings, such as local senior welfare centers, community health centers, apartments, housing complexes, and outpatient clinics to minimize selection bias. Each center recruited participants using quota sampling stratified by age (70–74, 75–79, and 80–84 years, in a 6:5:4 ratio) and sex (men and women, in a 1:1 ratio), considering the age distribution of the older Korean adult population and the prevalence of frailty by age groups [27]. A sample size of 3,014, planned for this study to establish a community-based frailty cohort, was targeted according to the power analysis results, of which 3,014 was sufficient to identify risk factors and predictors of frailty and to differentiate between health outcomes associated with frailty. Therefore, the inclusion criteria for KFACS participants were as follows: individuals aged 70–84 years, residing within the community, with no anticipated plans to relocate outside the three specified neighboring towns within the next two years, no known communication barriers, and no prior diagnosis of dementia.

**Fig. 1:**
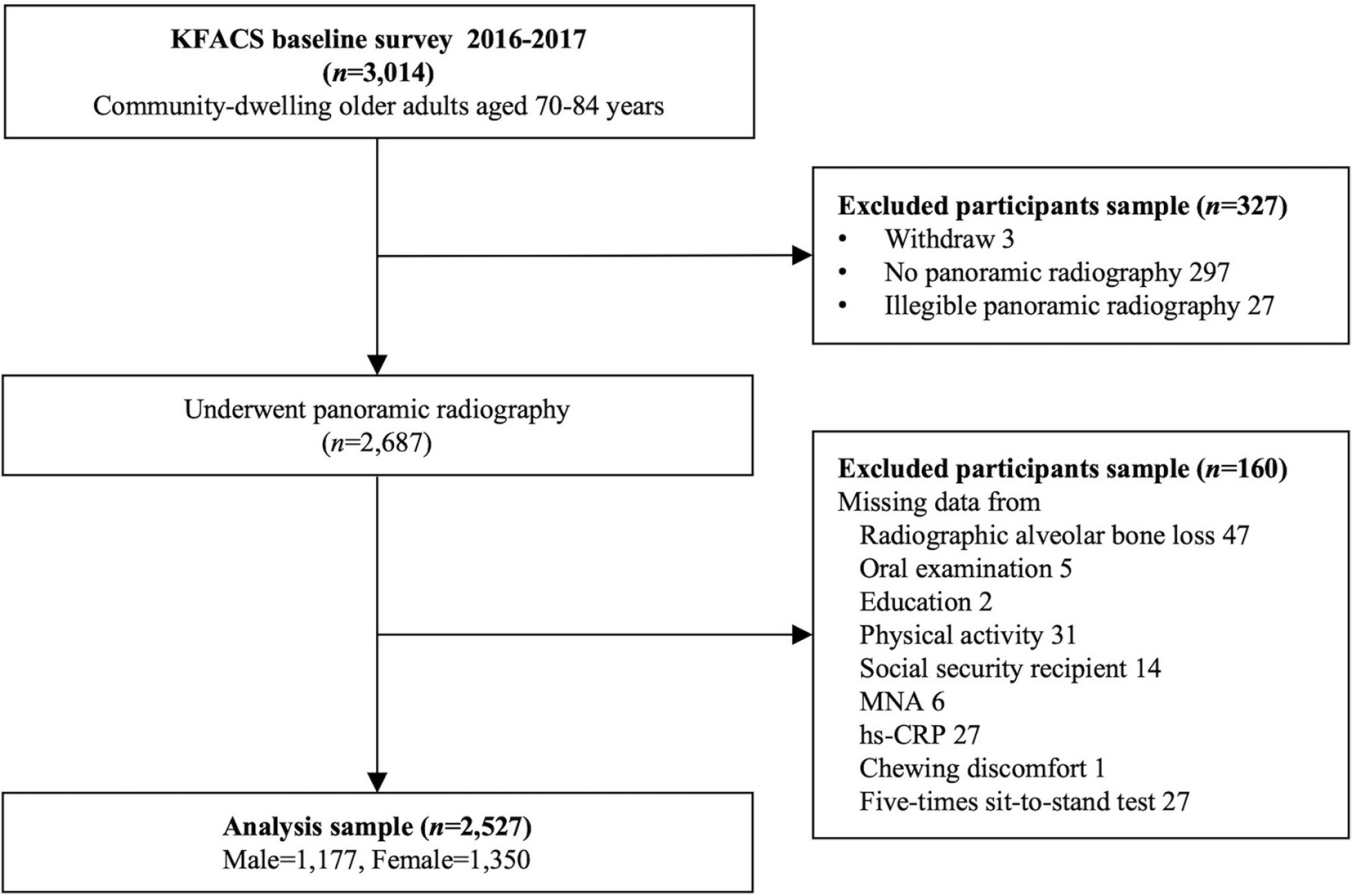
Flowchart of the study population

Among a total of 3,014 participants in the baseline KFACS, those who withdrew (*n* = 3) or had no panoramic radiography (*n* = 297) or illegible panoramic radiography (*n* = 27) were excluded. Of the 2,687 participants who underwent panoramic radiography, those with missing data on radiographic alveolar bone loss (RABL), oral examination, education, physical activity, social security recipient status, the Korean version of the Mini-Nutritional Assessment Short Form, high-sensitivity C-reactive protein (hs-CRP), chewing discomfort, and 5TSTS (*n* = 160) were also excluded. Accordingly, this study included 2,527 participants (Fig. 1). Ethical approval for the KFACS protocol was obtained from the Clinical Research Ethics Committee of Kyung Hee University (Institutional Review Board [IRB] number: 2015-12-103). The current investigation was deemed exempt from further review by the Clinical Research Ethics Committee of Kyung Hee University Medical Center (IRB number: 2024-08-011), which was conducted in compliance with the guidelines of the Declaration of Helsinki. All participants provided written informed consent.

### Assessment of the number of functional teeth

In the KFACS, panoramic radiography was performed instead of oral examination to assess dental status, including the number of functional teeth, presence of dental prostheses, and periodontitis. As the KFACS is a nationwide multicenter longitudinal study, panoramic radiography is unavoidable for data on dental status in a standardized manner across every center, owing to limited time and resources [26, 28]. Panoramic radiography provides a comprehensive anatomical view of the teeth, maxilla, mandible, temporomandibular joints, and related facial structures in a single image. This approach allowed for a holistic assessment of oral conditions, contributing to the accuracy and reliability of our research findings. Moreover, it has been validated as a reliable screening tool for periodontitis [29]. To ensure consistency, panoramic radiographs were assessed by dentists who were trained and calibrated at a single study center using standardized criteria that supported the reliability of the readings. We informed the participants in advance about the potential for radiation exposure, including oral panoramic radiography, and obtained their informed consent. The radiation dose received during dental panoramic radiography ranges 5–49 µSv, with a mean effective dose of 22.16 ± 15.66 µSv, which is lower than the dose typically received during air travel [30].

The number of functional teeth was examined using panoramic radiography, with assessments conducted by trained and calibrated professionally registered dentists. Functional teeth were defined as the remaining natural teeth with visible crowns, with or without restorations, prosthetic pontics, and dental implants [19]. Root rests and severely decayed teeth were excluded and masticatory third molars were included as functional teeth. The number of functional teeth ranged from 0 to 32. Previous studies have suggested that having ≥ 20 teeth is necessary for normal masticatory function [11, 12]. Therefore, participants were categorized into two groups based on the number of functional teeth: < 20 and ≥ 20.

### Assessment of physical function

Handgrip strength was defined as the maximum handgrip strength in kilograms after measuring each hand twice using a handgrip dynamometer (T.K.K.5401; Takei Scientific Instruments Co., Tokyo, Japan). The participants were instructed to stand with their feet shoulder-width apart, arms outstretched away from the body, elbows fully extended, and shoulders and wrists in a neutral position. The participants rested for 3-min rest to ensure complete recovery after the first measurement. The usual gait speed over 4 m was measured using an automatic timer (Gaitspeedometer; Dynamic Physiology, Daejeon, Korea) with acceleration and deceleration phases of 1.5 m. Participants were instructed to walk at their usual pace. The test was performed twice with no rest time, and the average value was used. The 5TSTS assessed how quickly the participants could stand up and sit down five times. The participants were instructed to fold their arms across their chest, not use them during the test, start from the first seated position, and finish in the last standing position [31]. The duration of the 5TSTS was recorded. The SPPB consists of three standing balance measures, 5TSTS and usual gait speed. Balance measures evaluate the ability to hold tandem, semi-tandem, and side-by-side standing positions for at least 10 s each. Each test is scored from 0 (unable to complete a task) to 4 (highest level of performance), with a maximum total score of 12 [32]. Each physical function was divided into two categories according to the Asian Working Group for Sarcopenia (handgrip strength, men: 28 kg [< 28 and ≥ 28 kg], women: 18 kg [< 18 and ≥ 18 kg]; gait speed, 1.0 m/s [< 1.0 and ≥ 1.0 m/s]; 5TSTS, 12 s [< 12 and ≥ 12 s]; SPPB, 9 [≤ 9 and > 9 scores]) [33].

### Covariates

We obtained information on sociodemographic factors (age, sex, body mass index (BMI), education level, living alone, and social security recipient status), lifestyle (smoking status, alcohol consumption), health condition (hs-CRP and present medications), and oral health (tooth brushing frequency in a day, oral examination during the last year, and self-reported chewing discomfort). Low physical activity was assessed using the International Physical Activity Questionnaire in a population-based Korean survey of older adults and was defined as the lowest 20% of energy expenditure, corresponding to < 494.65 kcal per week for men and < 283.50 kcal per week for women [34]. Nutritional status was assessed using the Korean version of the Mini-Nutritional Assessment Short Form [35]. The participants were divided into three groups: normal (12–14), risk of malnutrition (8–11), and malnourished (0–7) [36]. If the Mini-Mental State Examination Korean version score was < 24, participants were classified as having cognitive dysfunction [37]. Depressive symptoms were defined as having the Korean version of the Short Form Geriatric Depression Scale score of ≥ 6 [38]. Comorbidity was defined as the presence of two or more of the following chronic diseases: hypertension, myocardial infarction, cardiovascular disease, congestive heart failure, cerebrovascular disease, asthma, chronic obstructive pulmonary disease, diabetes mellitus, dyslipidemia, osteoarthritis, rheumatoid arthritis, or osteoporosis. The dental prostheses used in the present study were pontics, implants, and crowns. Participants were divided into those without dental prostheses and those with one or more dental prostheses. Periodontitis was diagnosed using the RABL, which was defined as the distance from the cemento-enamel junction to the alveolar bone crest, measured on the mesial and distal sides of all teeth at 0.1 mm level [39, 40]. The ImageJ program (1.48 software, National Institutes of Health, Bethesda, MD, USA) was used to measure the lengths in the panoramic radiographs. Periodontitis was classified into five categories as follows: normal (< 3 mm), mild periodontitis (two or more interproximal sites with 3 ≤ RABL < 4 mm), moderate periodontitis (two or more interproximal sites with 4 ≤ RABL < 6 mm), severe periodontitis (two or more interproximal sites with RABL ≥ 6 mm) and edentulism [39–41].

### Statistical analysis

The general characteristics of the study participants between the two groups categorized by the number of functional teeth (< 20 and ≥ 20) were compared according to sex using Pearson’s chi-square test for categorical variables and independent-samples Student’s t-test for continuous variables. The association between the number of functional teeth and physical function was assessed using a multivariate linear regression analysis. Multivariate logistic regression analysis was used to assess the association between < 20 functional teeth and low physical function after full adjustment for the following covariates: age, body mass index (BMI), education, living alone, social security recipient status, alcohol consumption, smoking status, physical activity, cognitive dysfunction, depressive symptoms, nutritional status, hs-CRP, comorbidity, number of present medications, dental prostheses, tooth brushing frequency, oral examination during the last year, self-reported chewing discomfort, and periodontitis. All statistical analyses were performed using IBM SPSS for (version 28.0 IBM Cop.). Statistical significance was set at *p* < 0.05.

## Results

### Characteristics of study population

Table 1 shows the characteristics of the study population. The mean age of the 2,527 participants was 76.5 ± 3.9 years. Overall, 34.5% of the total participants had < 20 functional teeth. Participants with < 20 functional teeth, both men and women, were generally older, had a lower BMI, and had lower education levels. They were also more likely to live alone, experience cognitive impairment, depressive symptoms, poor nutritional status, and rely on social security. These individuals tended to take more medications, lacked dental prostheses, brushed their teeth less often, had chewing discomfort, had not undergone an oral exam in the past year, and had a higher prevalence of severe periodontitis and edentulism, compared to those with ≥ 20 functional teeth (all *p* < 0.05). Men with < 20 functional teeth were more likely to smoke and have higher hs-CRP levels than those with ≥ 20 functional teeth (all *p* < 0.05), a trend not seen in women. Conversely, women with < 20 functional teeth also had a higher incidence of current alcohol consumption and low physical activity than those with ≥ 20 functional teeth (all *p* < 0.05), a pattern not observed in men. In both men and women, participants with < 20 functional teeth had lower handgrip strength, slower gait speed, longer 5TSTS duration, and lower SPPB scores compared to those with ≥ 20 functional teeth (all *p* < 0.001)

**Table 1:** Comparison of characteristics between the groups categorized by the number of functional teeth

Characteristics	Total (*n*=2527)	Men (*n*=1177)			Women (*n*=1350)		
		<20 functional teeth (*n*=406)	≥20 functional teeth (*n*=771)	*p*	<20 functional teeth (*n*=463)	≥20 functional teeth (*n*=887)	*p*
***Sociodemographic factors***							
Age (years)	76.5 (3.9)	78.0 (3.9)	76.2 (3.8)	<0.001	77.7 (3.8)	75.5 (3.6)	<0.001
BMI (kg/m^2^)	24.5 (3.0)	23.5 (2.9)	24.3 (2.9)	<0.001	24.5 (3.1)	25.0 (3.0)	<0.001
Education (<7 years)	46.3	48.3	40.7	0.013	60.7	42.6	<0.001
Living alone	22.9	12.6	7.0	0.001	41.0	32.0	<0.001
Social security recipient	7.1	8.9	4.7	0.004	12.7	5.5	<0.001
***Lifestyle***							
Current drinker	17.3	33.0	32.3	0.805	5.8	2.9	0.009
Current smoker	5.8	17.0	8.2	<0.001	1.7	0.7	0.070
Low physical activity	11.5	11.1	12.5	0.492	13.4	9.8	0.046
Cognitive dysfunction (MMSE-KC score <24)	22.8	23.9	11.3	<0.001	40.4	23.2	<0.001
Depressive symptoms (GDS score ≥6)	23.2	20.9	13.0	<0.001	38.2	25.3	<0.001
Nutrition status (MNA)				<0.001			<0.001
Normal (12–14)	84.4	79.1	87.7		77.1	87.7	
At risk of malnutrition (8–11)	14.6	18.7	11.8		21.6	11.5	
Malnourished (0–7)	1.0	2.2	0.5		1.3	0.8	
***Health condition***							
hs-CRP				0.003			0.494
<1.0 mg/L	64.1	55.9	65.6		63.9	66.5	
1.0-3.0 mg/L	26.4	30.8	25.6		27.4	24.5	
>3.0 mg/L	9.6	13.3	8.8		8.6	9.0	
Comorbidity (≥2)	53.6	41.1	43.6	0.420	64.1	62.5	0.542
Number of present medications	3.6 (3.0)	4.0 (3.4)	3.6 (3.1)	0.029	3.7 (3.0)	3.3 (2.8)	0.009
***Oral health***							
Dental prostheses (≥1)	67.2	46.8	77.3	<0.001	46.0	78.9	<0.001
Tooth brushing frequency (times/day)	2.4 (0.8)	2.2 (1.0)	2.4 (0.9)	0.003	2.4 (0.8)	2.5 (0.7)	0.021
No oral examination within 1-year	66.8	75.6	59.8	<0.001	79.3	62.3	<0.001
Self-reported chewing discomfort	47.6	58.9	36.3	<0.001	70.2	40.4	<0.001
Periodontitis (RABL)				<0.001			<0.001
Normal (<3 mm)	8.7	12.8	3.9		15.8	7.3	
Mild (3–4 mm)	14.6	6.4	12.7		11.4	21.5	
Moderate (4–6 mm)	39.5	23.9	43.2		30.2	48.3	
Severe (≥6 mm)	31.7	41.9	40.2		25.7	22.8	
Edentulism	5.5	15.0	0.0		16.8	0.0	
Number of functional teeth	20.3 (9.5)	8.5 (6.2)	26.5 (2.8)	<0.001	8.6 (6.5)	26.4 (2.6)	<0.001
Number of natural teeth	10.4 (8.2)	3.0 (4.3)	14.7 (7.2)	<0.001	2.7 (3.9)	14.0 (6.8)	<0.001
***Physical function***							
Handgrip strength (kg)	26.1 (7.5)	30.3 (6.1)	32.9 (5.7)	<0.001	20.3 (4.1)	21.2 (4.1)	<0.001
Gait speed (m/s)	1.1 (0.2)	1.1 (0.2)	1.2 (0.2)	<0.001	1.0 (0.2)	1.1 (0.2)	<0.001
5TSTS duration (s)	11.6 (4.2)	11.7 (4.3)	10.4 (3.0)	<0.001	13.2 (5.3)	11.8 (4.2)	<0.001
SPPB score (score)	10.7 (1.6)	10.7 (1.5)	11.3 (1.1)	<0.001	10.0 (1.9)	10.7 (1.5)	<0.001

Data are presented as mean (SD) for continuous variables and as % for categorical variables

Abbreviations: BMI, body mass index; MMSE-KC, Mini-Mental State Examination Korean version; MNA, Mini-Nutritional Assessment Short Form; hs-CRP, high-sensitivity C-reactive protein; GDS, global deterioration scale; RABL, radiographic alveolar bone loss; 5TSTS, Five-times sit-to-stand test; SPPB, Short Physical Performance Battery; SD, standard deviation

### Association between the number of functional teeth and physical function

Table 2 presents the association between the number of functional teeth and physical function in the multivariate linear regression models. In men, even after full adjustment, an increase in one functional tooth was associated with an increase in handgrip strength of 0.049 kg, gait speed of 0.002 m/s, and SPPB score of 0.019 (all *p* < 0.05). The time taken for 5TSTS was 0.033 s shorter for an increase in one functional tooth in men (*p* < 0.001) (Model 4). In women, an increase in one functional tooth was related to an increase in gait speed by 0.002 m/s and SPPB score by 0.018 (all *p* < 0.05) (Model 4). An increase in functional teeth by one tooth resulted in a corresponding decrease in the time taken for the 5TSTS by 0.036 s among women (*p* < 0.001) (Model 4). However, the association between the number of functional teeth and handgrip strength disappeared after adjusting for sociodemographic factors in women (Model 1).

**Table 2:** Association of the number of functional teeth with physical function at baseline according to sex

	Unadjusted		Model 1		Model 2		Model 3		Model 4	
Dependent variables	B	95% CI	B	95% CI	B	95% CI	B	95% CI	B	95% CI
**Men**										
Handgrip strength	0.144^***^	0.109, 0.178	0.080^***^	0.047, 0.114	0.061^***^	0.028, 0.095	0.058^***^	0.024, 0.091	0.049^**^	0.012, 0.086
Gait speed	0.005^***^	0.003, 0.006	0.003^***^	0.002, 0.005	0.002^**^	0.001, 0.004	0.002^**^	0.001, 0.003	0.002^*^	0.000, 0.003
5TSTS duration	-0.068^***^	-0.089, -0.048	-0.047^***^	-0.068, -0.026	-0.035^**^	-0.057, -0.014	-0.035^**^	-0.056, -0.013	-0.033^**^	-0.057, -0.010
SPPB score	0.034^***^	0.026, 0.042	0.025^***^	0.017, 0.033	0.020^***^	0.012, 0.028	0.020^***^	0.012, 0.028	0.019^*^	0.010, 0.028
**Women**										
Handgrip strength	0.059^***^	0.036, 0.082	0.014	-0.010, 0.038	0.002	-0.022, 0.026	0.001	-0.022, 0.025	-0.003	-0.030, 0.024
Gait speed	0.006^***^	0.004, 0.007	0.003^***^	0.002, 0.004	0.002^**^	0.001, 0.003	0.002^**^	0.001, 0.003	0.002^*^	0.000, 0.003
5TSTS duration	-0.075^***^	-0.101, -0.049	-0.052^***^	-0.080, -0.025	-0.032^*^	-0.059, -0.005	-0.032^*^	-0.059, -0.005	-0.036^*^	-0.067, -0.006
SPPB score	0.041^***^	0.032, 0.050	0.026^***^	0.017, 0.036	0.018^***^	0.009, 0.027	0.018^***^	0.008, 0.027	0.018^***^	0.008, 0.029

*p < 0.05, **p < 0.01, ***p < 0.001

Model 1: adjusted for age, body mass index, education, living alone, and social security recipient

Model 2: further adjusted for current drinker, current smoker, low physical activity, nutrition status, cognitive dysfunction, and depressive symptoms

Model 3: further adjusted for hs-CRP, comorbidity, and number of present medications

Model 4: further adjusted for dental prostheses, tooth brushing frequency, no oral examination within 1-year, self-reported chewing discomfort, and periodontitis

Abbreviations: B, regression coefficient; CI, confidence interval; 5TSTS, five-times sit-to-stand test; SPPB, Short Physical Performance Battery; hs-CRP, high-sensitivity C-reactive protein

### Association of < 20 functional teeth with low physical function

**Fig. 2:**
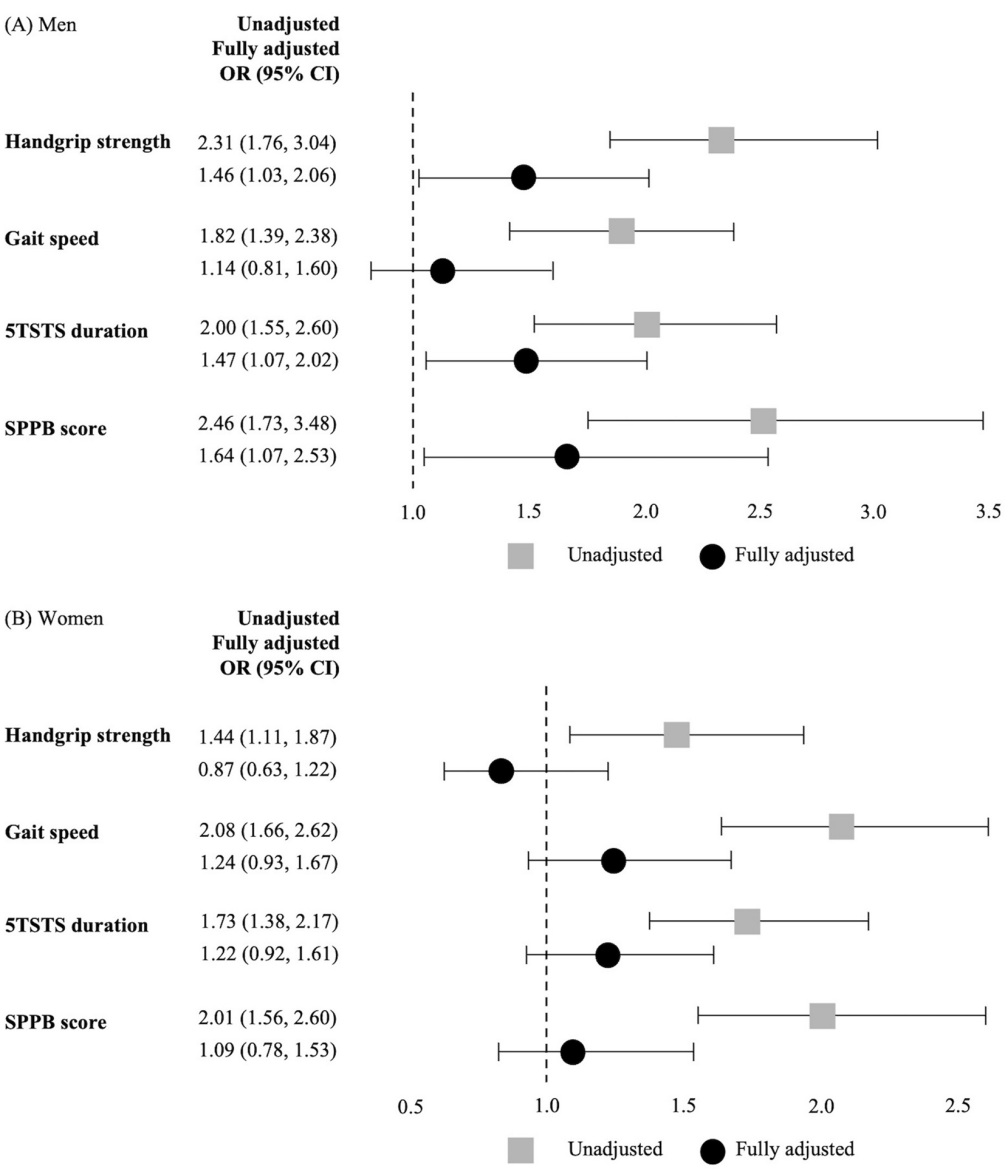
Forest plot of the multivariable logistic regression models. (**A**) Association between the < 20 functional teeth and low physical function in men. (**B**) Association between the < 20 functional teeth and low physical function in women. Fully adjusted for age, BMI, education, living alone, social security recipient, alcohol consumption, smoking status, physical activity, nutrition status, cognitive dysfunction, depressive symptoms, hs-CRP, comorbidity, number of present medications, dental prostheses, tooth brushing frequency, oral examination during the last year, self-reported chewing discomfort, and periodontitis. Abbreviations: OR, odds ratio; CI, confidence interval; 5TSTS, five-times sit-to-stand test; SPPB, Short Physical Performance Battery; BMI, body mass index; hs-CRP, high-sensitivity C-reactive protein

The effects of < 20 functional teeth on each physical function in the unadjusted and fully adjusted models are shown in the forest plot (Fig. 2). In men, participants with < 20 functional teeth had a higher risk of low handgrip strength than those with ≥ 20 functional teeth after full adjustment (95% Confidence Interval [CI]: 1.03–2.06). The association between < 20 functional teeth and slow gait speed in men did not persist, even after full adjustment (95% CI: 0.81–1.60). Men with < 20 functional teeth still had a higher likelihood of long 5TSTS duration (95% CI: 1.07–2.02) and low SPPB scores (95% CI: 1.07–2.53) than those with ≥ 20 functional teeth in the fully adjusted model. In women, < 20 functional teeth were not significantly associated with low handgrip strength, slow gait speed, long 5TSTS duration, and low SPPB scores after full adjustment (Fig. 2).

## Discussion

This study investigated the association between number of functional teeth and physical function after adjusting for various covariates in community-dwelling older Korean adults. Our findings showed that a higher number of functional teeth was associated with faster gait speed, shorter 5TSTS duration, and higher SPPB score in both men and women but was associated with higher handgrip strength only in men. Furthermore, having < 20 functional teeth was associated with low handgrip strength, long 5TSTS duration, and low SPPB scores after full adjustment for sociodemographic, lifestyle, health conditions, and oral health in older men. Our study confirms that having sufficient functional teeth is associated with maintaining adequate physical function in older adults.

Our results indicated a significant positive linear association between the number of functional teeth and handgrip strength only in men. In women, the association between the number of functional teeth and handgrip strength disappeared after adjusting for sociodemographic factors, including age, BMI, education, living alone, and social recipient status, whereas it remained significant in men. To explore the sex differences, we performed subgroup and interaction analyses. The participants were grouped based on age (stratified by the age of 80 years). In men, there was no significant interaction between the subgroups and the number of functional teeth (*p* for interaction = 0.256). In contrast, in women, there was a significant interaction between age and the number of functional teeth (*p* for interaction = 0.049). In other words, age has a significantly greater effect on handgrip strength in women, not in men. The findings from the subgroup analysis explain why the association between the number of functional teeth and handgrip strength was not significant in women in Model 1 adjusted for sociodemographic factors, including age (Table 2). This finding suggests that sociodemographic variables may mediate or diminish the strength of this relationship among women.

In this study, a higher number of functional teeth was associated with a faster gait speed, shorter 5TSTS duration, and higher SPPB scores in both men and women. This is consistent with a previous study in which older adults with more teeth had better physical function and lower disability [23]. Gait speed, 5TSTS, and SPPB are physical performance tests used to assess lower limb function and mobility. The partial or complete loss of occlusion causes a decline in leg extensor power or balance, indicating the ability to move in daily life [42]. Reduced lower extremity strength is associated with a decrease in balance and getting up from a seated position [43]. Specifically, the masticatory muscular system and dentoalveolar ligaments play important roles in maintaining the posture of the head and body during proprioception of the mandibular system [44]. Overall, poor dental occlusion leading to loss of functional teeth may disturb normal balance and lower limb function.

Multivariate logistic regression analysis was conducted to determine whether having < 20 functional teeth was associated with low physical function. In our study, having < 20 functional teeth was significantly associated with a higher risk of low handgrip strength, long 5TSTS duration, and low SPPB scores only in men after full adjustment. A previous study of older Korean adults, which showed that having 0–9 remaining teeth was associated with a higher risk of having low handgrip strength compared to having ≥ 20 remaining teeth only in men [17]. A previous study has reported that having ≥ 20 teeth significantly protects against a low SPPB score compared with having no teeth [44]. Furthermore, a previous study on older British men reported that losing functional dentition and becoming edentulous were related to a decline in 5TSTS speed [45]. Targeting the maintenance of a minimal number of teeth can play a role in early identification of future deteriorated oral and general health among middle-aged and older adults, as tooth loss is a final indicator of oral and general health [12]. Taken together, our results suggest that maintaining at least 20 functional teeth is associated with normal physical function, such as handgrip strength, 5TSTS, and SPPB, in older men.

Sex differences in the association between the number of functional teeth and physical function may occur because women are more susceptible to sociodemographic factors than men. Previous studies have reported that a lower educational level is associated with fewer remaining teeth and lower physical function scores in women [46, 47]. Moreover, overweight older Korean women, with a BMI of 25.0–27.5 kg/m^2^, have been shown to have reduced mortality risk [48]. Estrogen secreted by subcutaneous fat prevents a decline in muscle strength in postmenopausal women [49, 50]. Because estrogen affects muscle fiber protein synthesis and proteolysis in women, reduced estrogen levels in postmenopausal women cause a decrease in muscle mass, which is associated with a decrease in muscle strength [50]. In our study, women had higher BMI and lower education levels than men (all *p* < 0.05, data not shown). Therefore, sociodemographic factors such as BMI and education, rather than number of functional teeth, may have a greater influence on physical function in older women.

Two potential causal pathways have been identified through which functional teeth may be associated with poor physical function. First, a decline in masticatory ability leading to loss of functional teeth causes older adults to avoid natural foods that are difficult to chew, such as raw vegetables, meat, and fresh fruits [10]. This deficient dietary intake can exacerbate age-related changes in body composition such as a higher proportion of fat mass and a lower proportion of muscle and bone mass [51, 52]. It affects nutritional status, leading to impaired physical function indirectly [53, 54]. Second, periodontitis, which is characterized by chronic inflammation within the oral cavity, leads to serious conditions that ultimately result in tooth loss [55]. Prolonged systemic inflammation resulting from periodontitis is associated with impaired physical function through sarcopenia and systemic damage [55–57]. We hypothesized that a pathway could explain the significant association between functional teeth and physical function, including oral health, even after full adjustment. This may be due to the reactive oxidative species in severe periodontitis. A previous study reported that severe periodontitis results in reactive oxygen species via an inflammatory response in the oral cavity [58]. In addition, a recent study has reported that oxidative stress can trigger apoptosis and senescence, which are accompanied by impaired physical performance [59].

This study has some limitations. First, this cross-sectional study did not demonstrate a causal relationship. Second, our findings may not generalize to more dependent populations such as hospital inpatients or nursing home residents. Our study population consisted of community-dwelling older adults who participated independently and voluntarily in a baseline survey. Therefore, extrapolating these results to dependent older adults requires caution. Lastly, we performed panoramic radiography instead of oral examination to assess dental status. Owing to the absence of clinical oral examinations, assessment of the periodontal tissue condition and use of dentures was unavailable. Compared with oral examinations, oral panoramic radiography has a degree of distortion and cannot assess the periodontal condition. In addition, each of the 10 centers participating in the KFACS study used a different instrument. Further studies assessing dental status through oral examinations are needed. Nevertheless, the strength of this study is that it is the first to examine the association between functional teeth and physical function in older Korean adults.

## Conclusions

Our study demonstrates that a low number of functional teeth is independently associated with poor physical function in community-dwelling older Korean adults. We observed sex-related differences in the association between the number of functional teeth and handgrip strength. Furthermore, having < 20 functional teeth was associated with a higher risk of poor physical function in men, including handgrip strength, 5TSTS, and SPPB. Our results emphasize the importance of maintaining an adequate number of functional teeth to preserve physical function in older adults. These findings also provide evidence supporting prosthetic rehabilitation as a preventive strategy for increasing the number of functional teeth.

## Data Availability

The data used in the study is not publicly available, but the data used and/or analyzed during the current study are available from the corresponding author on reasonable request.

## Abbreviations

CI: Confidence interval
BMI: body mass index
hs-CRP: high-sensitivity C-reactive protein
KFACS: Korean Frailty and Aging Cohort Study
MMSE-KC: Mini-Mental State Examination Korean version
MNA: Mini Nutritional Assessment Short-Form
OR: Odds ratio
RABL: Radiographic alveolar bone loss
SD: Standard deviation
SPPB: Short Physical Performance Battery
5TSTS: five-times sit-to-stand test
